# Incorporating Cold-Air Pooling into Downscaled Climate Models Increases Potential Refugia for Snow-Dependent Species within the Sierra Nevada Ecoregion, CA

**DOI:** 10.1371/journal.pone.0106984

**Published:** 2014-09-04

**Authors:** Jennifer A. Curtis, Lorraine E. Flint, Alan L. Flint, Jessica D. Lundquist, Brian Hudgens, Erin E. Boydston, Julie K. Young

**Affiliations:** 1 U. S. Geological Survey, California Water Science Center, Eureka, California, United States of America; 2 U. S. Geological Survey, California Water Science Center, Sacramento, California, United States of America; 3 U. S. Geological Survey, California Water Science Center, Placer Hall, California, United States of America; 4 University of Washington, Department of Civil and Environmental Engineering, Seattle, Washington, United States of America; 5 Institute for Wildlife Studies, Arcata, California, United States of America; 6 U. S. Geological Survey, Western Ecological Research Center, Thousand Oaks, California, United States of America; 7 U. S. Department of Agriculture, Wildlife Services, National Wildlife Research Center and Utah State University, Wildland Resources Department, Logan, Utah, United States of America; University of Oxford, United Kingdom

## Abstract

We present a unique water-balance approach for modeling snowpack under historic, current and future climates throughout the Sierra Nevada Ecoregion. Our methodology uses a finer scale (270 m) than previous regional studies and incorporates cold-air pooling, an atmospheric process that sustains cooler temperatures in topographic depressions thereby mitigating snowmelt. Our results are intended to support management and conservation of snow-dependent species, which requires characterization of suitable habitat under current and future climates. We use the wolverine (*Gulo gulo*) as an example species and investigate potential habitat based on the depth and extent of spring snowpack within four National Park units with proposed wolverine reintroduction programs. Our estimates of change in spring snowpack conditions under current and future climates are consistent with recent studies that generally predict declining snowpack. However, model development at a finer scale and incorporation of cold-air pooling increased the persistence of April 1^st^ snowpack. More specifically, incorporation of cold-air pooling into future climate projections increased April 1^st^ snowpack by 6.5% when spatially averaged over the study region and the trajectory of declining April 1^st^ snowpack reverses at mid-elevations where snow pack losses are mitigated by topographic shading and cold-air pooling. Under future climates with sustained or increased precipitation, our results indicate a high likelihood for the persistence of late spring snowpack at elevations above approximately 2,800 m and identify potential climate refugia sites for snow-dependent species at mid-elevations, where significant topographic shading and cold-air pooling potential exist.

## Introduction

Previous studies document a general decline in snowpack over the last half century throughout the western United States [1], [2]. The loss of snowpack coincides with regional warming, and within California the warming trend is expected to continue with expected increases in mean annual temperatures of 2.2 to 8.3°C over the next 100 years [3]. Documented effects of regional warming in California’s alpine regions include: earlier onset of spring snowmelt [4]–[6], reduced summer base flows [7], [8], declines in snowpack volumes at mid-elevations [9], and migration of the rain to snow transition line to higher elevations [10].

In the midst of regional declines in snowpack previous studies documented a net increase in snowpack at elevations above 2,500 m within the southern Sierra Nevada [1], [11], [12]. This reversal from the regional decline in snowpack may be related to increases in atmospheric moisture due to the presence of warmer air masses capable of holding more water [2]. Increases in atmospheric moisture combined with adiabatic cooling can lead to increased snow accumulation at the highest elevations where winter and early spring temperatures remain below the temperature threshold for the transition from snow to rain, even under predicted regional warming.

**Figure 1:**
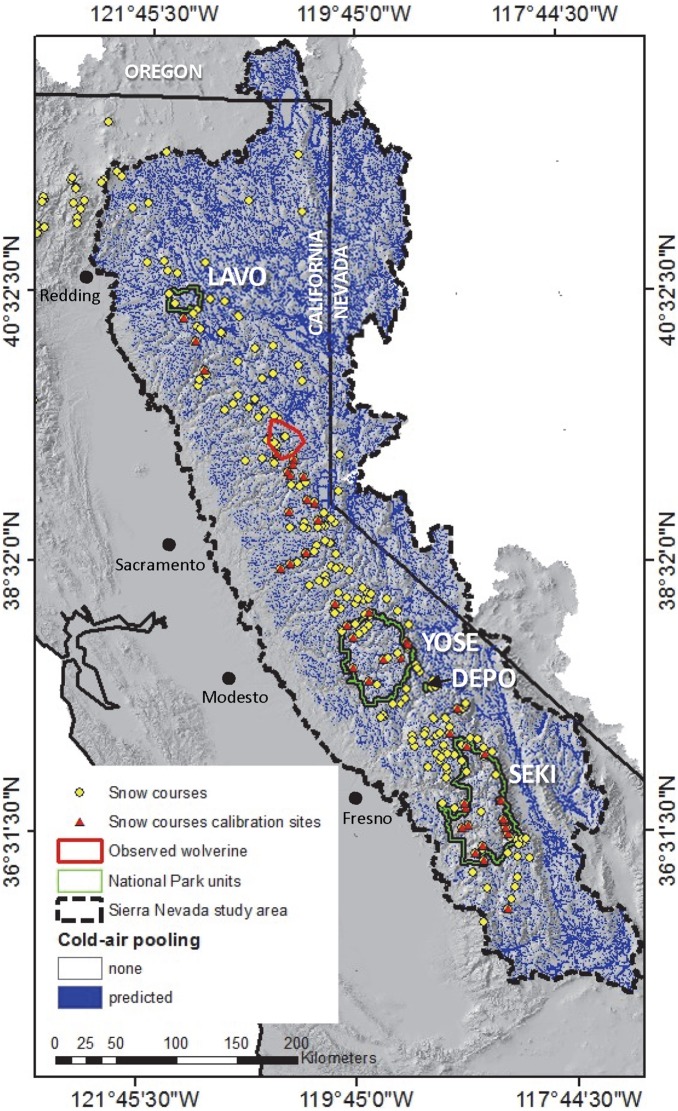
Study area map showing cold-air pooling potential. Map of Sierra Nevada Ecoregion showing the location of snow courses, snow model calibration sites, range of resident wolverine sitings, National Park units (Lassen Volcanic National Park, LAVO; Yosemite National Park, YOSE; Sequoia-Kings National Parks, SEKI; and Devils Postpile National Monument, DEPO), and cold-air pooling potential.

Due to its complex glaciated terrain the Sierra Nevada Ecoregion (Figure 1) is prone to cold-air pooling (CAP), a nocturnal atmospheric process that sustains cooler air temperatures in CAP–prone areas. On calm clear nights air in contact with the ground cools due to radiative energy loss. Being denser than the free atmosphere at the same elevation, this colder-denser air becomes decoupled and sinks into topographic depressions. CAP occurs where cooled air collects on the landscape. CAP generally occurs in concavities and areas cut off from the free atmosphere, typically along flat valley bottoms in mountainous terrain with valley constrictions [13]–[21]. Exposed ridges and topographic convexities are not prone to CAP.

Due to the decoupling and sinking of colder denser air, CAP-prone areas may respond differently to predicted climate change than surrounding terrain. For example, CAP-prone snow covered sites in Idaho, Montana, and Wyoming, warm less rapidly than non-CAP sites located in exposed locations [22]. Furthermore, CAP may not only diminish the impacts of regional warming in high mountain valleys [23], [24] but, along with coincident topographic shading provided by surrounding terrain, CAP may actually delay snowmelt despite increases in air temperatures [6]. In this study we investigate the influence of CAP on air temperatures and snowmelt and assess whether incorporating CAP into future climate scenarios leads to snowpack persistence.

The trajectory of change in snowpack under future climates will undoubtedly impact snow-dependent ecology. Consequently, habitat-scale projections of future snowpack conditions are needed to provide information to support conservation and management decisions. In this study we use the wolverine (*Gulo gulo*) as an example species. Because the wolverine is an obligate carnivore with a northern circumpolar distribution [25] it’s range limits are strongly correlated with snow covered areas [26], [27]. Current research indicates that spring snowpack is a critical resource for providing suitable denning sites to support successful wolverine reproduction [28].

Historically, the Sierra Nevada wolverine population represented the southernmost extent of the species’ range, but wolverines were believed to be extirpated from this region by the 1920s [26], [29]–[32]. In 2008, a lone male wolverine (Figure 1), most likely an immigrant from Idaho, was photo-documented [33]. If a population of wolverines is re-established in the Sierra Nevada Ecoregion, it would provide population redundancy to this threatened species, which currently only occupies a small area of the northern Cascades and Rocky Mountains in the conterminous United States [26], [34]. Determining the feasibility of wolverine reintroduction to the Sierra Nevada Ecoregion requires investigation of potential suitable habitat that includes adequate spring snowpack under current and future climates.

We present a unique approach for developing spatially distributed estimates of snowpack under future climates. Currently, global climate models (GCMs) are developed at coarse spatial scales and future climate projections cannot represent fine-scale processes. It is well recognized that fine-scale modeling incorporates the effects of topographic heterogeneity and results in improved precipitation estimates [35], [36]. A fine-scale is further required to capture processes that control accumulation, melt, and sublimation of snow [37]. However, this is the first study that incorporates a fine-scale process such as CAP into projections of snowpack under future climates. Our methodology includes the definition and application of a temperature correction factor such that the role of CAP, in mitigating snowmelt and sustaining the depth and extent of snow covered areas, is incorporated into future projections of snowpack. We then present an example ecological application by investigating late spring snowpack conditions under historic, current and future climates and the presence of snow covered habitat in four National Park units with proposed wolverine reintroduction programs.

### Study Area

The study area encompasses 131,650 km^2^ of the Sierra Nevada Ecoregion (Figure 1). We further highlight results for four National Park units with proposed wolverine reintroduction programs. The park units include Yosemite (YOSE), Sequoia-Kings Canyon (SEKI), and Devils Postpile (DEPO) within the Sierra Nevada National Park Network and Lassen (LAVO) National Park from the Klamath Network. Elevations within the study area range from 40 to 4,415 m, with the highest elevations occurring in the southern Sierra Nevada. Under the current climate, mean annual precipitation ranges from 50 cm on the western edge of the study area to more than 150 cm at the highest elevations along the Sierra Nevada crest [38]. Precipitation is generated primarily by Pacific frontal systems that supply approximately 85% of annual precipitation between November and April.

Regions above 3,000 m were glaciated repeatedly throughout the Quaternary geologic period [39], [40]. Topographic shading and CAP allowed small glaciers in sheltered high mountain cirques to persist at elevations well below the regional permanent snowline defined at 4,500 m [41]. The persistence of permanent snowpack throughout the Sierra Nevada Ecoregion over geologic time scales and at relatively lower elevations highlights a distinct ability for producing and sustaining snowpack and snow covered habitat.

The glaciated terrain of the Sierra Nevada is uniquely suited for producing CAP due to characteristic valley morphologies. Fluvial and glacial valley profiles are typically concave, but glacial valley profiles are over-deepened with higher concavities and steeper headwater profiles [42], [43] and typically have wide flat valley bottoms and lower gradients at the glacial terminus [44], [45]. CAP is further influenced by valley constrictions that occur where wider U-shaped glacial valleys transition into narrower V-shaped fluvial valleys. Hanging valleys, located at the confluence of tributary and trunk streams, and stepped topography, formed due to an increase in cross sectional area to accommodate the added tributary stream discharge, are additional topographic complexities [41] that may increase the potential for CAP in glaciated terrain.

## Methods

We used a published regional water balance model (BCM) developed at a 270 m grid scale [46], to estimate historic (1951 to 1980), current (1981 to 2010), and future (2011 to 2100) snowpack conditions throughout the Sierra Nevada Ecoregion. The BCM employs a deterministic water-balance calculation and utilizes SNOW-17, a temperature index model [47], [48], to estimate monthly snow accumulation and snow melt. The 270 m grid required spatial downscaling of available air temperature and precipitation data. The downscaling approach [49] preserves and enhances topographic effects.

Initially the adequacy of the snowmelt calculation was evaluated based on the timing of streamflow in high elevation subbasins [46]. The snow model calibration was refined in this study by comparing model results to snow covered area (SCA) at the basin scale and to measured snow water equivalents (SWE) at 45 spatially distributed calibration sites. We selected 1998 to 2009 as the calibration period and used the root-mean squared error (RMSE) and the sum of differences (SUMDIFF) to compare model results with measured snowpack data.

The calibration process was iterative such that we started with literature values for the adjustable snow parameters [50], which were changed accordingly to improve the goodness-of-fit between measured and modeled SWE. We visually assessed spatial correlations with the presence of permanent snowpack and the spatial distribution of snow covered areas. The calibration required the snow model to produce persistent snowpack (SWE>0) at locations with permanent snowpack (http://glaciers.research.pdx.edu/) and reasonable distributions of snow cover area, which we assessed by comparing the spatial extent of modeled snow cover area with published estimates [51]. Modeled snow covered area was compared to the gap-filled daily fractional snow cover area [51], estimated using MODIS satellite images (http://zero.eng.ucmerced.edu/snow/csnwis/), for dates reflecting winter accumulation (February) and spring snowmelt (May) conditions from 2000–2003.

Modeled monthly SWE was compared to measured monthly SWE at 45 snow course calibration locations (data available from the California Data Exchange at http://cdec.water.ca.gov/). There are 360 snow courses within California, and we selected 45 representative stations within the study region (Figure 1) that were spatially distributed over a range of elevations (1,325 to 3,460 m). Each station had records adjusted by DWR for snow density differences and records that spanned December to May for the duration of the calibration period (1998 to 2009). We did not use available snow pillow data, which is measured at discreet locations, but rather calibrated the snow model solely using snow course data collected along transects, which better represent spatially averaged conditions.

A spatial representation of CAP potential for the study region (Figure 1) was estimated using an automated algorithm [23] that evaluates a digital elevation model (DEM) and identifies flat valley bottoms and concave areas where cold air pools are likely to form. The algorithm requires an estimate of the average peak-to-peak distance, defined as the distance between two peaks divided by a valley. We used a peak-to-peak distance of 1,500 m for the entire study region. Because the Sierra Nevada Ecoregion covers a large geographic extent, a correction for diverging valleys where cold air drains rather than pools in a given location [23], was not applied.

Gridded air temperature data from the PRISM Climate Group (http://prism.oregonstate.edu) capture the CAP process in the historic record by including a vertical layer weighting function [35]. Since CAP is a nocturnal process that reduces minimum air temperatures and cold-air pools are dissipated by mid-day, maximum temperatures are not impacted. We evaluated the extent to which the PRISM data represents CAP by comparing minimum monthly air temperatures at 243 snow course stations located in CAP and non-CAP areas spatially distributed throughout the study region (Figure 1). A temperature correction factor was then defined as the mean difference between monthly minimum air temperatures estimated in CAP-prone and non-CAP areas during the primary snow accumulation and melt season (December to May). The correction factor represents a spatial and temporal mean estimated using 243 snow course locations distributed throughout the study region over a range of representative elevations (1,265 to 3,490 m) and temporally averaged from 1896 to 2009.

Because GCM projections do not include the effects of CAP we adjusted the downscaled monthly air temperature data in CAP-prone areas by subtracting the temperature correction factor to incorporate the CAP process and then generated monthly gridded snow maps for future climatic conditions. We necessarily assumed that the magnitude and frequency of CAP under future climates will remain unchanged and applied the same temperature correction to all future climate datasets.

Changes in snow-cover habitat were evaluated using the wolverine as an example species. We defined the presence of suitable wolverine habitat based on an April 1^st^ threshold depth of snowpack. April 1^st^ represents the midway point between February 15^th^ and May 15^th^ which is the average period during which female wolverines typically give birth, raise and then graduate kits such that they are no longer dependent on their snowpack dens [28], [52]. April 1^st^ snowpack is therefore a suitable surrogate for determining available wolverine breeding and denning habitat. A conservative threshold for April 1^st^ snow depth was defined at 1 meter, which equates to an April 1^st^ SWE of 400 mm based on a snow density of 2.5 g/cm^3^ (http://cdec.water.ca.gov/snow/misc/density.html).

Four projections, defined in the Fourth Assessment Report (AR4) of the United Nation’s Intergovernmental Panel on Climate Change [53], were used in this study. The four projections were selected to portray a range of future climate conditions. Although updated projections were recently released in the Fifth Assessment Report [54], the fine-scale resolution necessary for this study required that we use available downscaled AR4 scenarios [49].

We selected two GCMs and two emissions scenarios capable of simulating the distribution of monthly temperatures and the strong seasonal cycle of precipitation that exists in the region [3]. Cayan and others [55] describe the four projections in detail for California. The two GCMs are the Parallel Climate Model (PCM) developed by the National Center for Atmospheric Research and the Department of Energy [56], [57] and the Geophysical Fluid Dynamics Laboratory CM2.1 model (GFDL) developed by National Oceanic and Atmospheric Administration [58], [59]. The greenhouse gas emissions scenarios include A2, which represents “business as usual” with no reduction in emissions, and B1 which represents “mitigated emissions.” We refer to the four future climate scenarios as “GFDL-A2,” “GFDL-B1,” “PCM-A2,” “PCM-B1.” The GFDL scenarios represent “hot-dry” future conditions and the PCM scenarios represent “warm-wet” conditions.

## Results

### Model Performance

Comparison of measured data with initial model runs indicated that we were generally underestimating snowpack throughout the study region. Increases in snow accumulation and decreases in snowmelt were achieved by adjusting snow parameters iteratively to improve the goodness-of-fit between measured and modeled SWE. We started with maximum and minimum melt factors of 3.6°C and 2.2°C and a minimum temperature threshold of 4.5°C for the transition from rain to snow. Final melt factor values of 1.8°C and 1.3°C and a minimum temperature threshold of 6°C generated more snow throughout the study region, allowed permanent snowpack to occur in regions where it naturally occurs, and improved the goodness-of-fit between measured and modeled SWE. Because the model operated on a monthly time-step, these values differ greatly from those required when SNOW-17 is run at finer temporal resolutions.

**Figure 2:**
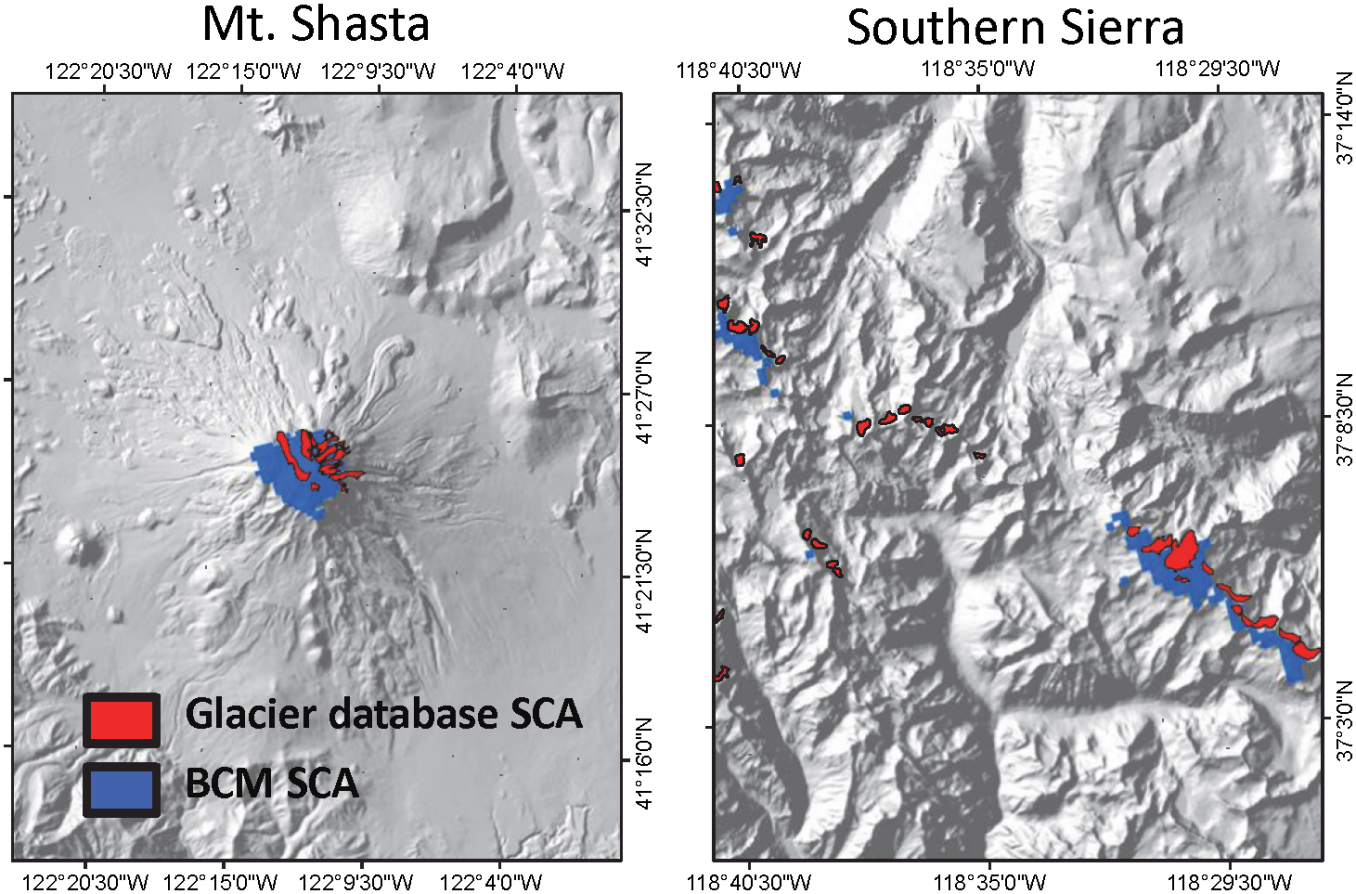
Visual comparison between modeled snow covered area and permanent snowpack locations. Comparison of snow covered area (SCA) simulated using the Basin Characterization Model (BCM) for September 2009 and areas of persistent snowpack for Mount Shasta, located in the northern study region, and along the southern Sierra Nevada crestline within the headwaters of the San Joaquin and Kings River watersheds.

**Figure 3:**
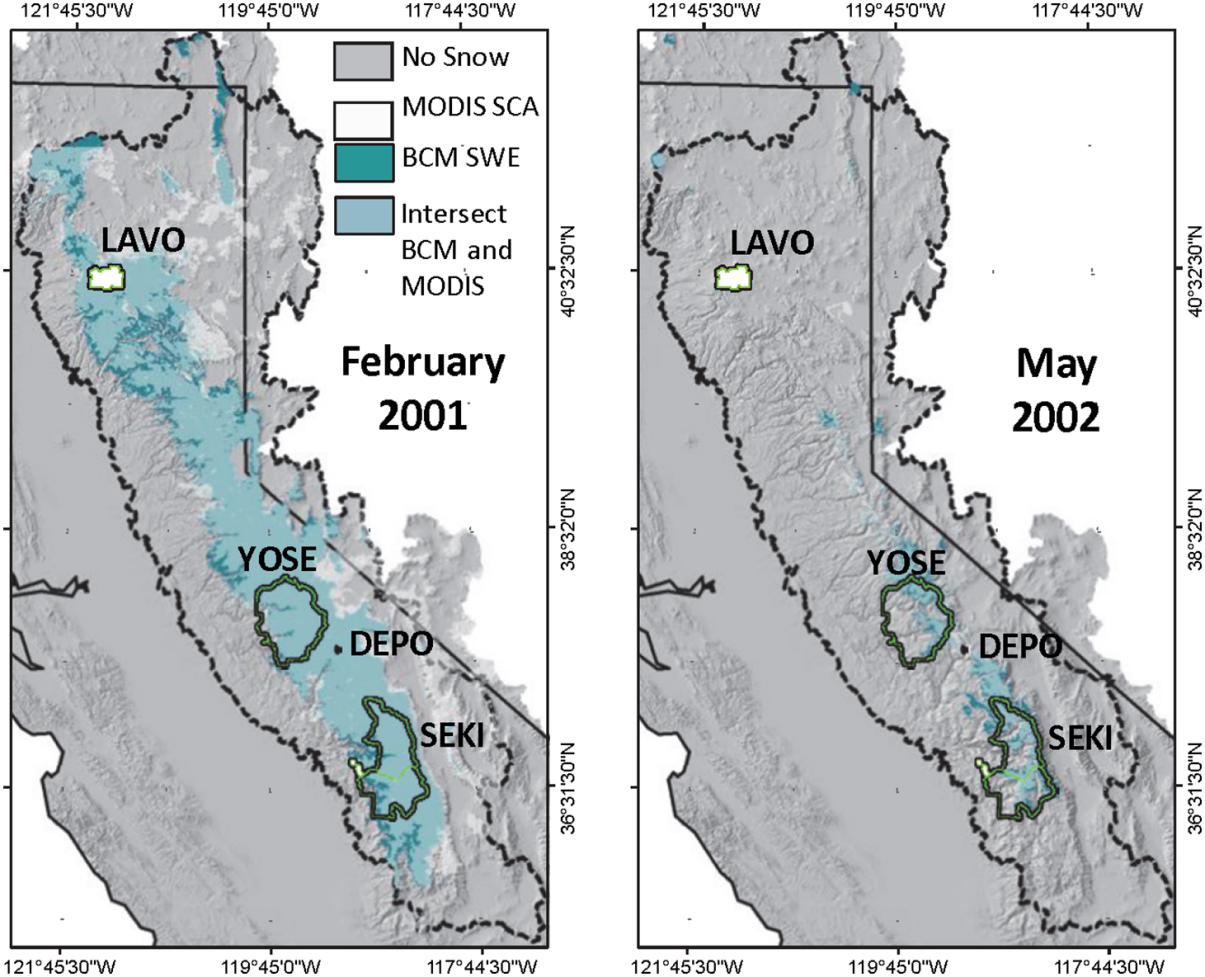
Visual comparison between modeled snow covered area and satellite imagery. Comparison of snow water equivalent (SWE) simulated with the Basin Characterization Model (BCM) and MODIS snow covered area (SCA) for February 2001 and May 2002.

We assessed the spatial accuracy of modeled SWE by visual comparison with permanent snowpack zones and satellite imagery depicting actual snow covered area. The spatial extents of modeled SWE consistently intersected locations with permanent snowpack (Figure 2). Similarly, modeled SWE consistently intersected estimates of snow covered area [51] determined using MODIS satellite imagery (Figure 3).

Our final regression analysis of measured versus modeled SWE at the 45 calibration sites resulted in an r^2^ of 0.416, RMSE of 328 mm, and SUMDIFF of −8.44E+04 mm indicating modeled SWE values reasonably approximate measured SWE. Separate regression analyses specifically assessed the influence of elevation (Table 1) and latitude (Table 2) on the relation between measured and modeled SWE values. When the results are categorized into three elevation classes, modeled SWE is most accurate at higher elevations above 2,500 m (RMSE = 238 mm) and least accurate at mid-elevations between 2,130 to 2,500 m (RMSE = 432 mm) where the rain to snow transition typically occurs (Table 1). When the results are separated by latitude, the RMSE indicates modeled SWE is more accurate in the northern region (RMSE = 277 mm) in comparison to the southern region (RMSE = 412 mm) but the SUMDIFF indicates the magnitude of the differences was higher in the northern region in comparison to the southern region (Table 2). These are expected results as the density of meteorological stations is higher, and thus PRISM data better represent the climate, in the Northern Sierra where the topography is less complex.

**Table 1:** Elevation effects on goodness-of-fit parameters for modeled snow water equivalents during the calibration period from 1998 to 2009.

	Root Mean Squared Error (mm)	Sum of Differences (mm)
Average	328	−8.44E+04
<2130 m	361	9.51E+03
2130 to 2500 m	432	−6.64E+04
>2500 m	238	−2.75E+04

**Table 2:** Latitude effects on goodness-of-fit parameters for modeled snow water equivalents during the calibration period from 1998 to 2009.

	Root Mean Squared Error (mm)	Sum of Differences (mm)
Average	328	−8.44E+04
Northern Region >/ = 38.5°	277	−6.71E+04
Southern Region <38.5°	412	−1.73E+04

Our analysis of monthly air temperatures from 1896 to 2009 indicates average monthly minimum air temperatures (AVG-TMIN) in CAP-prone locations are 1.6°C lower than AVG-TMIN in non-CAP locations during the primary snow accumulation and melt season from December to May (Table 3). Based on AVG-TMIN differences, the effects of CAP appear to be greatest at the beginning of the snow accumulation season from December to February, when the AVG-TMIN difference is ∼1.8°C. CAP declines moderately throughout the spring and the AVG-TMIN difference by May is ∼1.3°C. Based on these data, a spatially and temporally averaged monthly air temperature correction factor of 1.6°C was used to simulate CAP under future climate scenarios. The temperature correction was applied to air temperature datasets for the four future climate scenarios to incorporate the effects of CAP.

**Table 3:** Comparison of mean minimum air temperatures from 1896 to 2009 for snow courses with and without the potential to produce cold-air pooling (CAP).

	Minimum Air Temperature (°C)						
	Dec	Jan	Feb	Mar	Apr	May	Average
Non-CAP Sites (N = 106)	−6.05	−6.86	−6.94	−5.75	−3.77	0.25	−4.85
CAP-prone Sites (N = 137)	−7.85	−8.73	−8.75	−7.34	−4.97	−1.07	−6.45
Temperature differencebetween Non-CAP and CAPsites	1.80	1.87	1.81	1.59	1.20	1.32	1.60

See Figure 1 for station locations.

**Figure 4:**
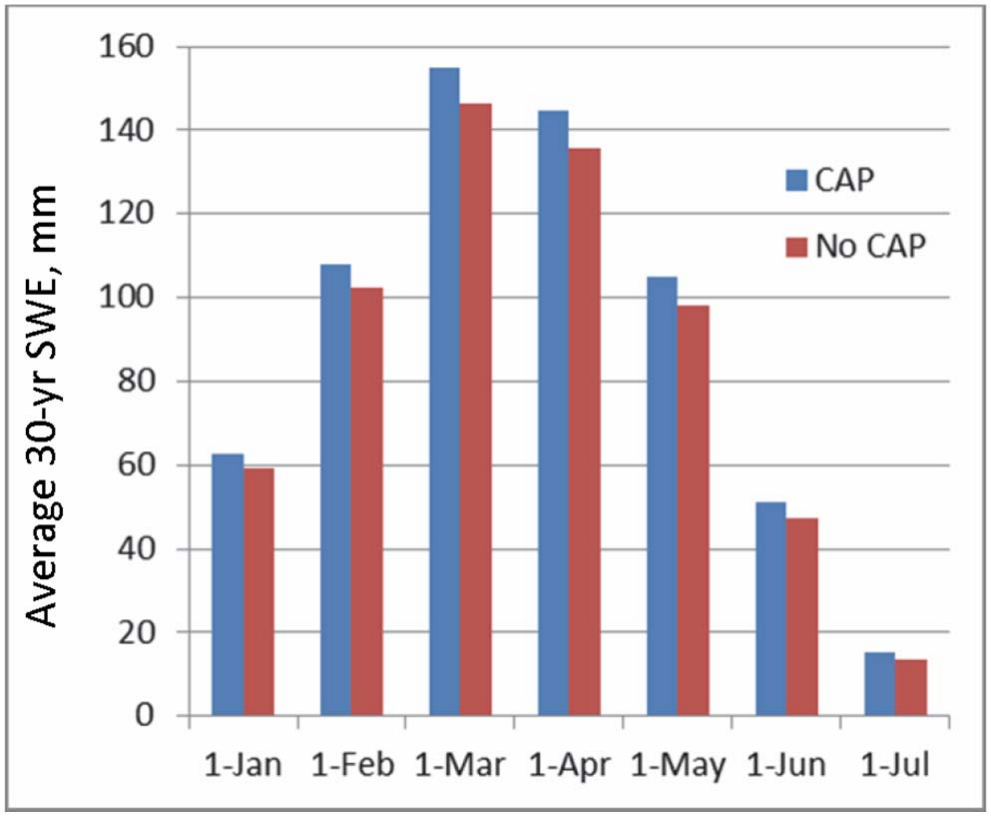
Influence of cold-air pooling on simulations of regional snow water equivalents. Monthly snow water equivalent (SWE) spatially averaged over the entire Sierra Nevada Ecoregion for the early-century period (2011–2040) using GFDL-A2, showing increases in SWE related to the use of a temperature correction factor (−1.6°C) to adjust minimum air temperatures and simulate cold-air pooling (CAP).

**Figure 5:**
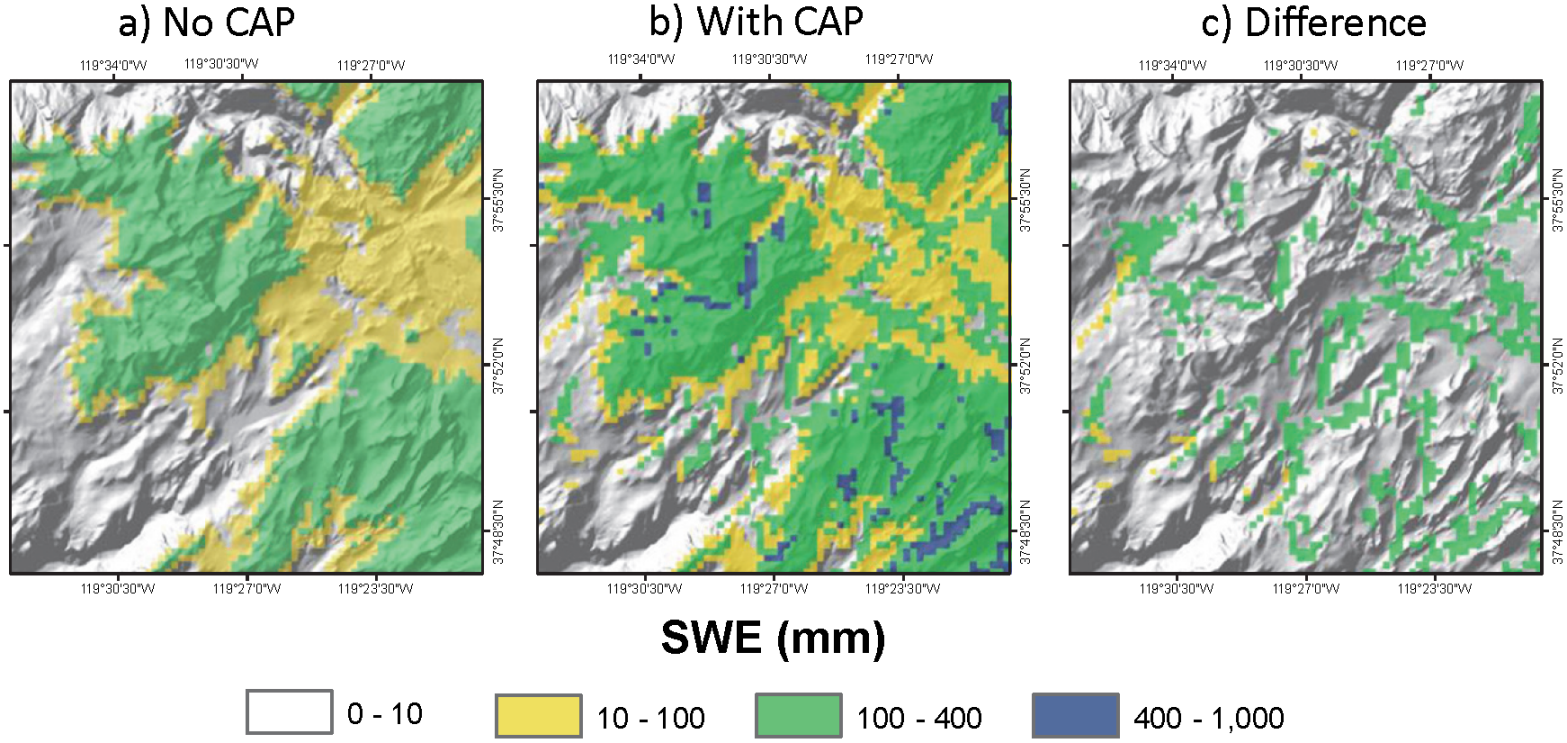
Influence of cold air pooling on persistence of snowpack at a finer scale. Comparison of June snow pack conditions shown for the headwaters of the Tuolumne River estimated using the worst case (GFDL-A2) future climate projection. Snowpack was simulated a) without an adjustment for cold-air pooling (CAP) and b) with a temperature correction factor (−1.6°C) to adjust air temperatures and simulate cold-air pooling (CAP). Panel c) shows the difference in the spatial extent and depth of SWE achieved by incorporating CAP into the future climate projection.

Incorporating CAP into future climate projections increased SWE for all snow dominated months (January to July). The increase in April 1^st^ SWE, averaged over the entire study region, was 6.5% and ranged from 5.5% to 11.1% for the pessimistic GFDL-A2 mid-21^st^ century projection (Figure 4). We compared the 6.5% simulated effect of CAP on April 1^st^ SWE to measured snowpack data collected at the 45 calibration sites from 1998 to 2009. The average April 1^st^ SWE was 690 mm at the non-CAP locations and 742 mm at the CAP-prone locations. The 52 mm or 8% increase in April 1^st^ SWE is comparable to the 6.5% simulated increase. An example of modeled SWE without CAP (Figure 5a) and modeled SWE with simulated CAP (Figure 5b) demonstrates the effect of CAP on the depth, spatial extent, and persistence of snowpack into early summer (June). The difference map (Figure 5c) highlights potential climatic refugia identified by incorporating CAP into future climate projections. Even for the most pessimistic mid-century scenario (GFDL-A2) simulation of CAP increased early summer SWE in CAP-prone areas by 100 to 400 mm.

### Estimating April 1^st^ Snowpack

We used the model results to quantify changes in spring snowpack under historic (1951 to 1980), current (1981 to 2010), and future (2011 to 2100) climates. Future climate results are separated into three 30-year projections: early-century (2011 to 2040), mid-century (2041 to 2070), and late-century (2071 to 2100). Additional results are focused on estimates of April 1^st^ SWE, a surrogate variable suitable for characterizing wolverine breeding habitat and we highlight changes in April 1^st^ SWE for four National Park units with proposed wolverine relocation programs [34].

**Figure 6:**
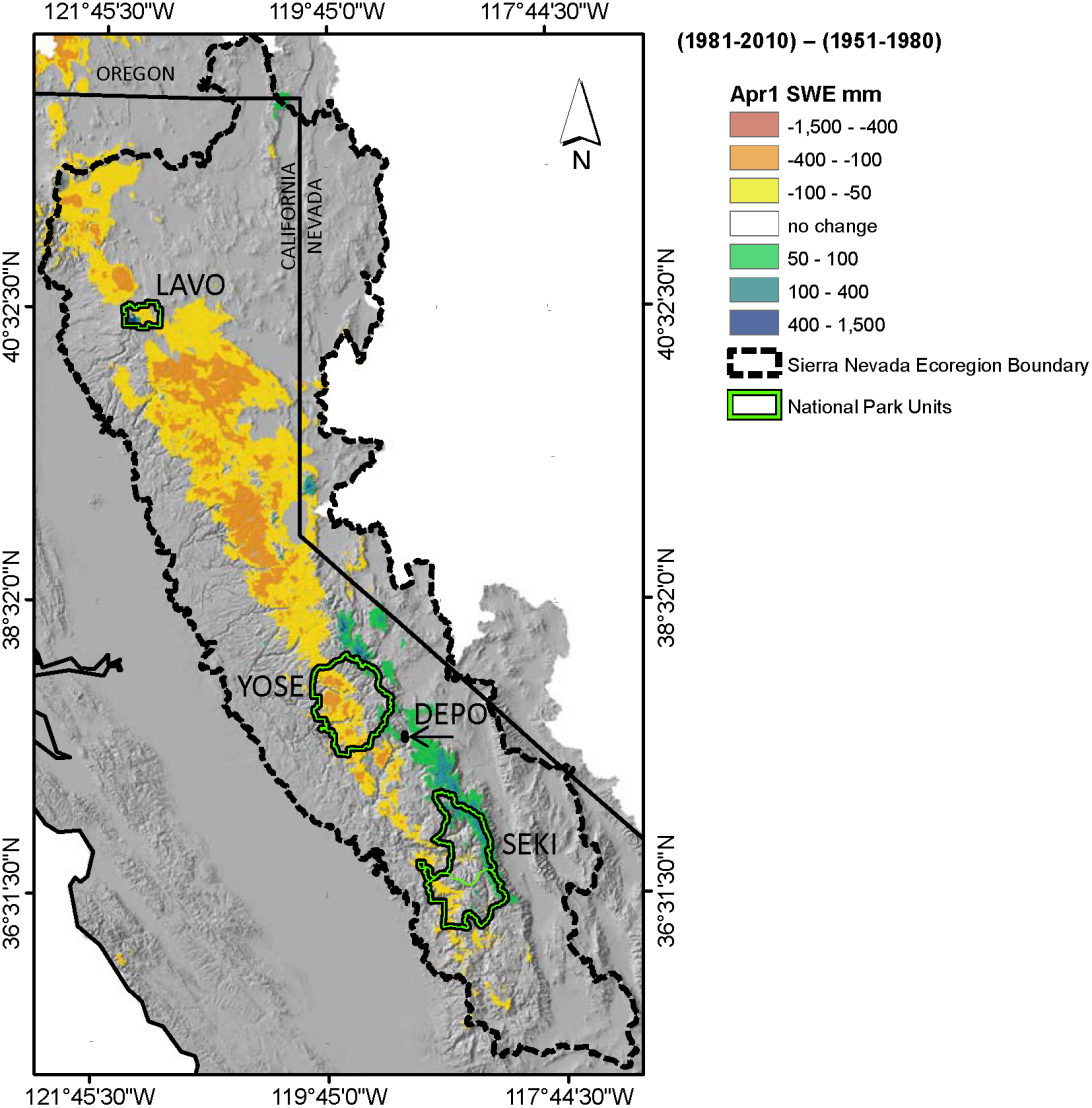
Historic changes in April 1^st^ snow water equivalents. A spatially distributed estimate of the change in April 1^st^ snow water equivalent (SWE) from historic (1951–1980) to current (1981–2010) climatic conditions.

We first present a spatial representation of the change in estimated April 1^st^ SWE by comparing historic and current climates (Figure 6). From a regional perspective, declining April 1^st^ SWE in the northern portion of the study area is balanced somewhat by increases in the southern Sierra Nevada. Declines in April 1^st^ SWE throughout the northern Sierra generally range from −50 to −400 mm whereas smaller magnitude increases of 50 to 100 mm were estimated for higher elevations along the southern Sierra Nevada crestline. The highest magnitude increases in April 1^st^ SWE were estimated for Lassen Peak located in the southwest corner of Lassen National Park (Figure 6). Generally, the net increase in April 1^st^ SWE under current climate conditions shown in Figure 6 occurred only at elevations above 2,800 m, a slightly higher elevation than reported in previous studies based on observational data [1], [12].

**Figure 7:**
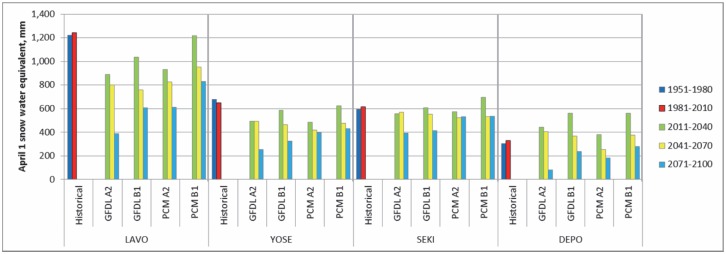
Changes in April 1^st^ snowpack under various climatic conditions in four National Park units. Simulated April 1^st^ snow water equivalent (SWE) spatially averaged across the boundaries of four National Park units for historic (1951–1980), current (1981–2010), and future (2011 to 2100) climatic conditions. The data, organized from left to right, show a worst case scenario (GFDL-A2), two moderate case scenarios (GFDL-B1 and PCM-A2) and a best case scenario (PCM-B1).

Figure 7 illustrates mean April 1^st^ SWE, spatially averaged across each National Park boundary, for historic, current, and future climate periods. Lassen National Park is located in the northern Sierra, where we estimated widespread declines in April 1^st^ SWE between historic and current climates (Figure 6); however Lassen Peak represents a relatively small high-elevation area with significant gains in April 1^st^ SWE under the current climate leading to an overall slight increase in April 1^st^ SWE between historic and current climates (Figure 7). The remaining three park units (Yosemite, Sequoia-Kings Canyon, and Devils Postpile) are located in the southern Sierra. We estimated declines in April 1^st^ SWE on lower elevation eastern slopes in the southern Sierra Nevada that were of a similar magnitude (−50 to −400 mm) to declines estimated in the northern region of the study area, but declines in the southern Sierra covered a smaller spatial extent. There are also significant areas with no net change in April 1^st^ SWE and extensive high elevation areas along the Sierra Nevada crest with increases in April 1^st^ SWE under current climatic conditions. This is especially evident in Devil’s Postpile, which is predominately located within a CAP zone, where future scenarios predict an increase in projected April 1^st^ SWE, relative to historic April 1^st^ SWE, until the mid-century (Figure 7).

Although projections of April 1^st^ SWE under future climates indicate Lassen may experience the largest declines among the National Park units, projections of April 1^st^ SWE remain higher for Lassen in comparison to the other park units for all four scenarios (Figure 7). Percent declines in Lassen’s April 1^st^ SWE for the late-century projection range from 33 to 69%, but abundant spring snowpack will likely persist in Lassen due to high elevation topography (Table 4). Percent declines in April 1^st^ SWE for the late-century projection for Yosemite and Devils Postpile were comparable to estimates for Lassen and range from 34 to 60% and 14 to 76% respectively; whereas projected percent declines for Sequoia-Kings Canyon were significantly less ranging from 13 to 36% (Table 4). Based on the late-century worst case projection (GFDL-A2) all National Park units may experience a 36 to 76% decline in April 1^st^ SWE, and even under the best case scenario (PCM-B1) the parks may experience a 13 to 34% decline (Table 4).

**Table 4:** Percent change in April 1st snow water equivalent from current climatic conditions (1981–2010) to early, mid and late 21st century climatic projections simulated for four National Park units located within the Sierra Nevada Ecoregion.

		LAVO				YOSE				DEPO					SEKI	
% changefrom1981–2010	GFDLA2	GFDLB1	PCMA2	PCMB1	GFDLA2	GFDLB1	PCMA2	PCMB1	GFDLA2	GFDLB1	PCMA2	PCMB1	GFDLA2	GFDLB1	PCMA2	PCMB1
2011–2040	−29	−17	−25	−2	−23	−9	−25	−4	35	71	16	71	−10	−2	−7	12
2041–2070	−36	−39	−34	−23	−24	−28	−35	−26	24	13	−22	15	−8	−10	−15	−14
2071–2100	−69	−51	−51	−33	−60	−49	−39	−34	−76	−28	−44	−14	−36	−36	−14	−13

**Figure 8:**
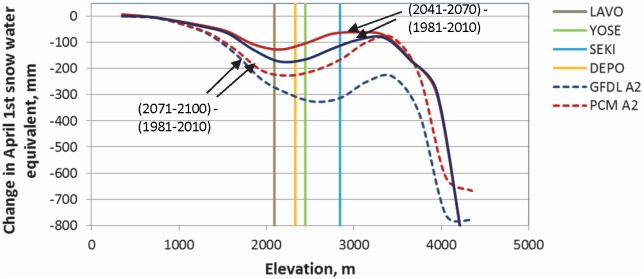
Influence of elevation on April 1^st^ snowpack throughout the Sierra Nevada Ecoregion. Change in April 1^st^ snow water equivalent (SWE) from current to mid-21^st^ century (solid lines) and late 21^st^ century (dashed lines) for elevation bands throughout Sierra Nevada Ecoregion. The location of National Park units are represented by their respective average elevations and future climate scenarios represent “business as usual” carbon emissions for warmer-wetter (PCM-A2) and warmer-drier conditions (GFDL-A2).

We further investigated changes in April 1^st^ SWE between the current, mid-century and late-century periods for the A2 scenario over a range of representative elevations (Figure 8). By mid-century most of the change in April 1^st^ SWE occurs at mid-elevations from approximately 1,800 m to 3,000 m. By late-century the change in April 1^st^ SWE moves upslope and is greatest between about 2,500 and 3,500 m. The mean elevation of each park unit is also shown in Figure 8 further indicating that Sequoia-Kings Canyon is projected to experience the greatest declines in April 1^st^ SWE by the late-century, while projections for Lassen indicate the least amount of change.

**Figure 9:**
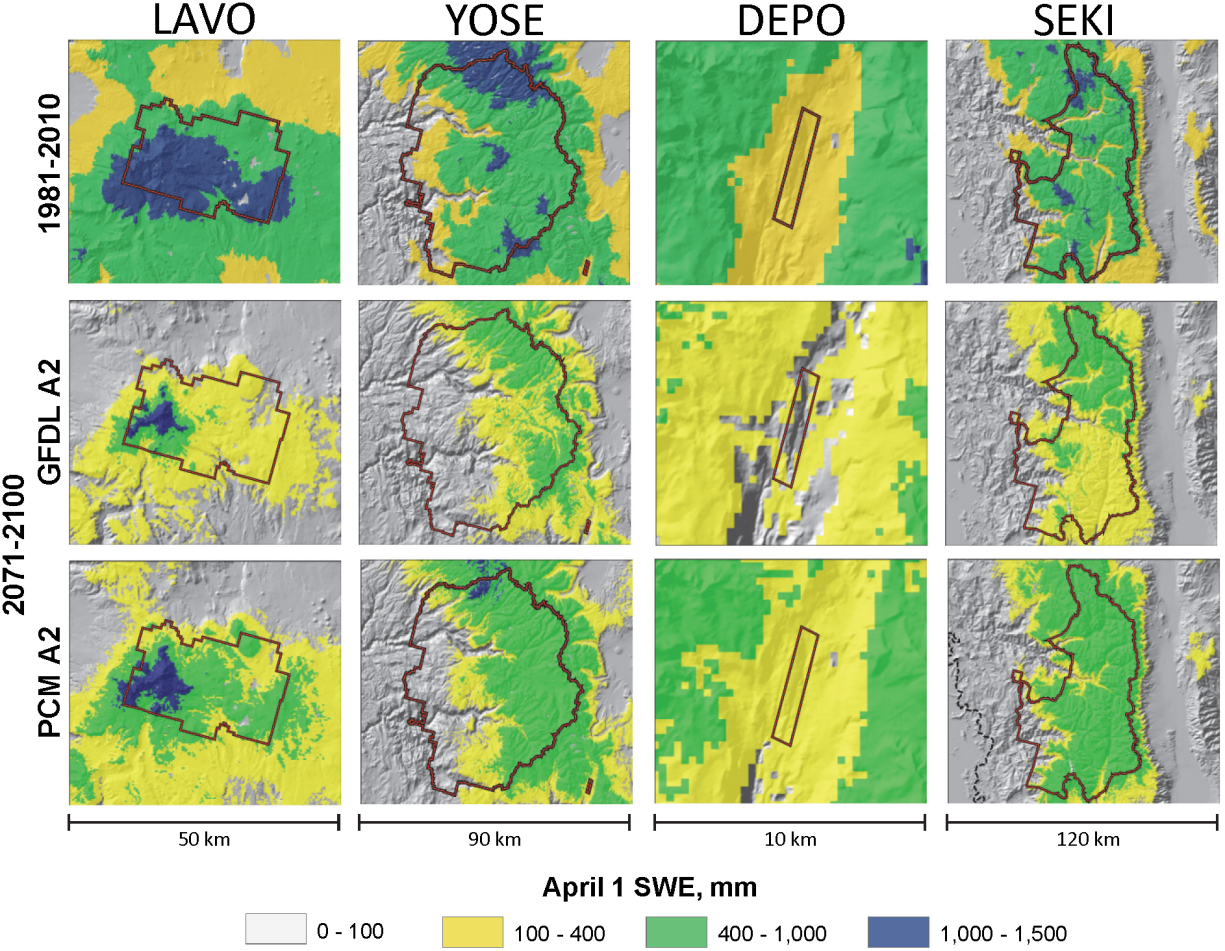
Simulated April 1^st^ snowpack under current and late-century climates for four National Park units. Detail of four National Park units in the Sierra Nevada Ecoregion showing April 1^st^ snow water equivalent (SWE) for current (1981–2010) and late 21^st^ century (2071–2100) climatic conditions. Late-century scenarios represent “business as usual” carbon emissions for warmer-wetter (PCM-A2) and warmer-drier future climates (GFDL-A2).

Impacts of changing snowpack conditions on snow covered habitat within the four National Park units were evaluated using the wolverine as an example species. Figure 9 shows a spatial representation of April 1^st^ SWE for each park unit under current climate conditions and for two late-century projections. The threshold April 1^st^ snowpack condition that represents the presence of suitable breeding and denning habitat is defined at 400 mm of SWE. Under historic and current climates the spatially averaged April 1^st^ snowpack is above the 400 mm threshold for Lassen, Yosemite, and Sequoia-Kings Canyon but not for Devils Postpile; however Devils Postpile is generally surrounded by higher elevations with sufficient snowpack. The relatively drier GFDL-A2 projection represents a worst case scenario and generally indicates larger magnitude declines in snowpack by the late-century in comparison to the wetter PCM-A2 projection (Figure 9) for all four National Park units. The worst case scenario (GFDL-A2) indicates average April 1^st^ snowpack will not meet the 400 mm threshold at any of the park units by the end of the 21^st^ century but substantial areas within and around the parks will be adjacent to potential higher elevation wolverine habitat that could be used for breeding and denning. The best case scenario (PCM-B1) indicates that the 400 mm April 1^st^ SWE threshold will be met in Lassen, Yosemite, and Sequoia-Kings Canyon by the end of the 21^st^ century. The “drier-mitigated emissions” scenario (GFDL-B1) indicates the April 1^st^ threshold will be met in Lassen and Sequoia-Kings Canyon. Under the “wetter-business as usual” scenario (PCM-A2) we estimate the wolverine habitat threshold will be met in Lassen, Yosemite, and Sequoia-Kings Canyon.

Under the two “business as usual” scenarios projected late-century snow covered areas in Lassen, above the 400 mm SWE threshold, represent about 30% of the total park area under the drier GFDL-A2 scenario whereas about 80% of Lassen’s snow covered areas exceed the threshold under the wetter PCM-A2 scenario. Notably, both “business as usual” scenarios indicate high elevation regions in Lassen will continue to maintain deep (>800 mm SWE) April 1^st^ snowpack. In Yosemite and Sequoia-Kings Canyon snow cover areas above the 400 mm threshold represent about 60% of the total park area in the wetter PCM-A2 scenario, but only about 20% for the drier GFDL-A2 scenario. Projections for Devils Postpile indicate no snow covered areas above the 400 mm SWE threshold within the park boundary under either late-century “business as usual” scenario but surrounding hillslopes provide snow covered areas with suitable SWE under the wetter scenario, while the zone with suitable SWE decreased and moved upslope under the drier scenario (Figure 9).

## Discussion

Results from the calibrated snow model presented here provide a spatial representation of snowpack for a vast regional area over a 150-year time period. The spatially and temporally averaged RMSE for the calibrated model is 328 mm, indicating estimated SWE values are typically within ∼0.3 m of measured data. The SUMDIFF estimates indicate SWE is generally underestimated throughout the study area, resulting in conservative projections of SWE and potential wolverine breeding and denning habitat. The modeled SWE values include uncertainties associated with the PRISM approach to spatially distribute measured climatic data, variability related to the spatially continuous model parameterization, and potential errors associated with measured snowpack at snow course calibration sites. Future improvements to the snow model could be realized by utilizing site-specific calibrations or latitudinal variations in parameterization as recommended by Raleigh and Lundquist [58].

Previous work showed that April 1^st^ SWE is better correlated to temperature than to precipitation in the northern Sierra Nevada [2] indicating regional warming will significantly impact the persistence of spring snowpack in the northern regions of the study area. Our results for current and future climates confirm this assertion. Conversely, the same study showed no correlation of April 1^st^ SWE with temperature at lower elevations but some correlation at higher elevations within the Southern Sierra Nevada [2]. Because April 1^st^ SWE is better correlated to precipitation in the Southern Sierra Nevada an increase in the accumulation of snowpack may occur at cooler high elevation sites where deeper snowpack has a higher potential to persist into late spring and early summer even under warmer climates. Conversely, regional warming without an increase in precipitation may result in large decreases in snowpack even at the coolest and highest elevations. If predictions of increased precipitation under future climates occur, our results indicate a high likelihood for the persistence of late spring snowpack at elevations above approximately 2,800 m. Our analysis of CAP also identifies a zone of potential climate refugia for snow-dependent species at mid-elevations where significant topographic shading and CAP potential exist.

Our estimates of changes in spring snowpack conditions under future climates are consistent with recent studies that generally predict significant declines. When future projections are compared to current conditions [11], [56], [61], earlier studies predict the highest reduction in snowpack volumes at low to mid-elevations (1,000 to 3,000 m). Figure 8 shows a comparison of the change in April 1^st^ snowpack, between current and worst case mid-century and late-century projections, for elevation bands throughout the study region. Our results indicate large magnitude changes in April 1^st^ SWE at the highest elevations, but the trajectory of change reverses at mid-elevations (2,000 to 3,500 m), where the National Park units are located, indicating mid-elevations may represent a zone of climatic refugia with an increased resilience leading to the persistence of April 1^st^ snowpack. Lassen, located at the lowest mean elevation of 2,090 m, is projected to experience the largest change in April 1^st^ SWE, whereas Sequoia-Kings Canyon, the park unit located at the highest mean elevation of 2,850 m, is projected to experience the least change in April 1^st^ SWE (Figure 7 and Table 4).

We compared our results to two recent studies that characterize potential changes in available wolverine habitat under future climates [62], [63]. Notably, the two previous studies selected less conservative spring snowpack depths to characterize potential wolverine habitat and were developed at coarser spatial scales. The first study used the Community Climate System model [62], developed at a much coarser grid of 250 km, and used a 20 cm snow depth threshold that produced pessimistic results indicating large declines in available wolverine habitat under future climates. The second study used an ensemble climate model [63], developed at a 12 km grid, and used a 13 cm snow depth threshold that produced more optimistic results. We believe characterization of snow-dependent habitat requires finer-scale analysis that incorporates topographic heterogeneities and CAP. We used a 270 m grid and incorporated a temperature correction factor into future climate scenarios in CAP-prone areas. Because we were specifically interested in characterizing wolverine breeding habitat for potential species reintroduction [34] we selected a more conservative spring snowpack threshold at 1 meter (equivalent to 400 mm SWE) which is associated with successful denning and reproduction [28].

Our results identify potential climatic refugia for snow dependent species throughout the Sierra Nevada Ecoregion. Three National Park units (Lassen, Yosemite, and Sequoia-Kings Canyon) all have areas that lie above 2,800 m, an equilibrium elevation defined in this study, where snow accumulation is occurring under current climatic conditions. Although Devils Postpile is located in a valley prone to CAP, our results indicate that this park unit is located at an elevation that has not accumulated large historic snowpack volumes and generally does meet our threshold of late spring snowpack (400 mm SWE) under current or future climates. Since home ranges of female wolverines are typically much larger (200 to 300 km^2^) than the area of Devils Postpile (2.8 km^2^), the presence of snowpack in the surrounding forest lands (Figure 9) indicates that wolverines could use the park as part of their home range although use of the park for denning would be rare.

Implementation of habitat-scale modeling and incorporation of CAP into the future climate projections were dominant factors contributing to optimistic projections of wolverine breeding and denning habitat throughout the Sierra Nevada Ecoregion. Utilization of a 270 m grid preserved topographic heterogeneity such that valley bottoms prone to CAP, high elevation peak and ridges that provide topographic shading, and north facing slopes where late spring snowpack typically persists remained distinct and not subdued by the spatially averaging that occurs at coarser grid scales.

The temperature correction applied to simulate CAP under future climate projections increased simulated April 1^st^ SWE by 6.5% over the regional model domain and identified fine-scale climatic refugia where snowpack is predicted to be maintained into the late spring and early summer. CAP zones may also represent climatic refugia for other snow-dependent species including predator species such as the pine marten (*Martes americana*), which hunt small mammals that rely on winter snow cover for survival [64], and species such as ermine (*Mustella ermine*), long-tailed weasels (*Mustella frenata*) and snowshoe hare (*Lepus americanus*), that rely on seasonally variable coat color for camouflage [65]. Thus, water balance models used to inform habitat studies must utilize appropriate scales to represent topographic complexities and must begin to incorporate fine-scale processes such as CAP.

## Conclusions

Results from this study provide data necessary to assess management and conservation strategies for snow-dependent species throughout the Sierra Nevada Ecoregion. The snow model explicitly incorporates topographic heterogeneity and CAP to estimate temporal and spatial variability of snowpack and the presence of snow covered habitat. Our estimates of changes in spring snowpack conditions under future climates are consistent with recent regional studies that generally predict significant declines. Notably, model development at a habitat-scale and incorporation of CAP resulted in significant mitigation of snow loss at mid-elevations, which may represent a zone of climatic refugia with a higher resilience for sustaining patches of snow suitable for wolverine breeding and denning and other species dependent on snow cover. We could not directly test whether CAP improves future snowpack projections, but our results show that incorporation of CAP produced more optimistic spring snowpack projections in CAP-prone areas supporting our central thesis that CAP creates climatic refugia for species dependent upon late spring snowpack.

We acknowledge that this is a relatively simple approach and recognize that the occurrence of CAP under future climates requires the presence of clear skies and high pressure systems, which are governed by large scale atmospheric circulation that could change under future conditions. Current GCMs do a poor job of predicting fine-scale changes in atmospheric circulation; however we expect that the next generation of GCMs will be able to better represent changes in atmospheric circulation and the presence of high pressure systems, thereby enabling more rigorous predictions of the magnitude and frequency of smaller scale atmospheric processes such as CAP.

Regional warming under the current climate has resulted in a higher percentage of precipitation falling as rain rather than snow throughout Western North America. This phenomenon will likely continue under future climates and even a modest temperature increase may significantly alter snow accumulation and melt processes. However, the highest elevations in the southern Sierra Nevada accumulated snow under the current climate. If predictions of increased precipitation under future climates are realized, our results indicate a high likelihood for the persistence of late spring snowpack at elevations above approximately 2,800 m and identify climate refugia sites for snow-dependent species at mid-elevations where significant topographic shading and cold-air pooling potential exist.
