# Correction: Incorporating Cold-Air Pooling into Downscaled Climate Models Increases Potential Refugia for Snow-Dependent Species within the Sierra Nevada Ecoregion, CA

**DOI:** 10.1371/journal.pone.0124729

**Published:** 2015-04-07

**Authors:** Jennifer A. Curtis, Lorraine E. Flint, Alan L. Flint, Jessica D. Lundquist, Brian Hudgens, Erin E. Boydston, Julie K. Young

There is an error in the last sentence of the third to last paragraph in the Methods. The correct sentence is: A conservative threshold for April 1st snow depth was defined at 1 meter, which equates to an April 1st SWE of 400 mm based on a snow density of 0.4 g/cm3 (http://cdec.water.ca.gov/snow/misc/density.html).
